# Alexander the Great and West Nile Virus Encephalitis

**DOI:** 10.3201/eid0912.030288

**Published:** 2003-12

**Authors:** John S. Marr, Charles H. Calisher

**Affiliations:** * Virginia Department of Health, Richmond, Virginia, USA; Colorado State University, Fort Collins, Colorado, USA

**Keywords:** Alexander the Great, retrodiagnosis, West Nile virus, Babylon

## Abstract

Alexander the Great died in Babylon in 323 BC. His death at age 32 followed a 2-week febrile illness. Speculated causes of death have included poisoning, assassination, and a number of infectious diseases. One incident, mentioned by Plutarch but not considered by previous investigators, may shed light on the cause of Alexander’s death. The incident, which occurred as he entered Babylon, involved a flock of ravens exhibiting unusual behavior and subsequently dying at his feet. The inexplicable behavior of ravens is reminiscent of avian illness and death weeks before the first human cases of West Nile virus infection were identified in the United States. We posit that Alexander may have died of West Nile encephalitis.

Alexander the Great died in the ancient Mesopotamian city of Babylon, on June 10, of 323 BC (Figure). His death after a 2-week febrile illness (Table) has fascinated ancient scholars and contemporary medical investigators (*1*), who have posited various diagnoses based on sparse clinical information—a few recorded signs and symptoms. Retrodiagnoses have included poisoning and infectious as well as noninfectious diseases (*1*–*6*). After reviewing ancient accounts and modern theories, we have concluded that Alexander may have died of West Nile encephalitis.

**Figure:**
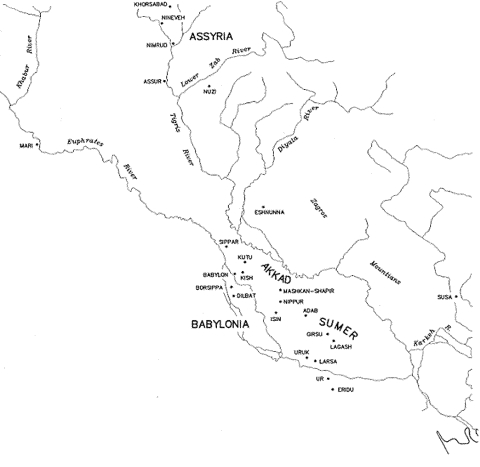
Map of Mesopotamia (present-day Iraq), including its capital, Babylon.

**Table:** Medical history and physical examination of Alexander the Great

Patient characteristics	Medical history	Clinical symptoms
Male Born in Macedonia 32 years of age Soldier Heavy drinking Frequent bathing Married to many wives One son	Ten years before death, traveled widely (Mediterranean, North Africa, and Middle East) Unexplained fever 5 years previously Penetrating right chest wound one year before final illness Onset of final illness May 29, 323 BC Death June 10, 323 BC	Escalating fever associated with chills Excessive thirst, diaphoresis Acute abdominal pain Single episode of back pain at of onset of fever Increased weakness leading to prostration with intermittent periods of energy Delirium Aphonia Terminal flaccid paralysis

## Previous Theories

### Poisons

Few poisons induce fever, and few of these were available in Alexander's time—except plant salicylates, which disturb temperature regulation; alkaloids, which interfere with perspiration; and ergot mycotoxins, which produce a subjective sensation of heat**.** Plutarch mentions that Aristotle (Alexander’s tutor) procured arsenic to poison Alexander (*7*). But plants, mycotoxins, and arsenic are not the likely causes of death since none would have caused the reported high, sustained fever.

### Infections

Alexander’s death occurred in late spring, upon his return to Babylon from the Indian subcontinent. Environmental conditions were unremarkable (*8*). Babylon, located on the Euphrates River (90 km south of present-day Baghdad), was bordered on the east by a swamp. Animals, including birds, were abundant (*9*), and arthropods were also likely present (available from: URL: http://www.ac.wwu.edu/~stephan/Animation/alexander.html). Diseases endemic to the area (present-day Iraq) (leishmaniasis, bubonic plague, hemorrhagic fevers) were not mentioned by chroniclers of Alexander’s death. Also not reported was illness among his troops, mainly Macedonians and local recruits. Descriptions of Alexander’s illness do not include common disease signs (e.g., rash, icterus, “thin blood,” vomiting, diarrhea or dysentery, hematuria, seizures).

Malaria, a diagnosis postulated by previous authors (*1*–*3*), occurred in Mesopotamia (*10*,*11*), and is common in today’s Middle East (*12*). Some of Alexander’s symptoms are compatible with malaria: continuous fever, chills, diaphoresis, prostration, myalgia, progressive weakness, stupor, diminished sensorium, delirium; however, dark urine, so called “black water fever,” or intermittent fevers were not reported. Today, most malaria in Iraq is due to *Plasmodium vivax* (*13*). Given Alexander’s travel history, had his illness been malaria, it would have been due to *P. falciparum*; however, absence of *P. falciparum*’s dramatic signature fever curve diminishes the possibility of malaria as a probable cause.

Typhoid fever and its complications have also been thoroughly considered (*1*). Alexander had a 2-week febrile illness culminating in terminal encephalopathy. As do encephalitis, endocarditis, pneumococcal pneumonia, psittacosis, rickettsial disease, and tularemia, typhoid causes sustained or continuous fever (*14*). The typical course of typhoid fever lasts one month. In fatal cases, death usually occurs at the end of week 2. Typhoid’s neurologic manifestations, which also include delirium and expressionless demeanor, are seen in week 3. Other signs include cough, diarrhea, “rose spots,” epistaxis, and bloody stool (*15*). None of these signs or other illnesses similar to Alexander’s were documented by Plutarch. Most other enteric infections have no neurologic sequelae and are generally self-limited. *Vibrio vulnificus* infection, which may cause fatal sepsis in heavy drinkers (as was Alexander), causes rapid death, accompanied by skin and muscle lesions and bleeding.

Other suggested diagnoses include *Schistosoma haematobium* infection (*4*), which causes painless hematuria; however, ectopic egg deposition may occur at any time, causing transverse myelitis, paralysis, and death (*16*). Exposure to cercariae produces pruritus and Katayama fever induces serum sickness (*4*), but symptoms include low grade fever and pruritic swellings, which were not reported in Alexander’s case. Some leptospirosis symptoms are consistent with Alexander’s illness; however, other classic leptospirosis signs (biphasic fever, calf or thigh pain, jaundice, hemorrhage, pulmonic involvement) were not reported. *Acanthamoeba* spp. (pathogenic free-living amoebae) and *Naegleria* spp. cause meningoencephalitis, which is acquired during bathing, an activity in which Alexander reportedly participated with compulsion. Acanthamoebae are cosmopolitan but prefer compromised hosts. Moreover, death from naegleriasis usually occurs within a week of onset, and encephalitis caused by acanthamoebae causes death only after a prolonged period of symptoms.

When Alexander’s clinical symptoms were listed on GIDEON (Global Infectious Diseases and EpidemiOlogy Network (*13*), influenza ranked highest (41.2% probability) on the list of differential diagnoses. While influenza could have killed Alexander, reports did not mention others becoming ill with similar symptoms. Lymphocytic choriomeningitis, an influenzalike illness followed by meningoencephalitis, is rare. Poliomyelitis can occur as an isolated case or as epidemic; its characteristics include fever, vomiting, severe myalgia, and prostration, as well as the early complication of flaccid paralysis, which has been postulated as another late sign in Alexander’s illness (*1*). This interpretation narrows the differential diagnosis to include poliomyelitis (see above), Guillain-Barré syndrome, and the encephalitides. (A list of the many other infectious diseases others have considered as well as additional, less likely candidates is available from the authors.)

## West Nile Fever and Encephalitis

West Nile fever was not considered by previous authors as cause of Alexander’s death, possibly because it has only recently emerged globally. West Nile virus (family *Flaviviridae*, genus *Flavivirus*), first isolated from a febrile patient in Uganda in 1937 (*17*), is one of many viruses causing encephalitis. Infection is marked by fever, encephalitis, or meningoencephalitis. Until the early 1990s, the virus was largely confined to Africa, Europe, and Asia. In 1941, an outbreak occurred in Tel Aviv, with no deaths reported. Over the next 60 years, seven outbreaks occurred in Israel and its environs (*18*). In 1957, during an outbreak in an army camp, a single case of encephalitis was recognized in a group of 300 soldiers (*19*). By 2000, a countrywide outbreak occurred, with a case-fatality rate of 8.4% (*20*). In 1999, West Nile virus was introduced to the United States, and 4,156 laboratory-confirmed human cases of infection (earliest onset of illness, June 10) occurred in 2002 (*21*). Median age in fatal cases was 72 years, although neurologic disease occurred in all cases. Also recognized in both fatal and nonfatal cases was flaccid paralysis in patients with encephalitis.

West Nile virus infections in vertebrates may have been occurring in the Middle East for centuries. Now the virus has spread to new areas of the world and to new populations and causes infection characterized by new signs and symptoms. In the 2000 epidemic in Israel, encephalitis occurred in nearly 59% of 417 human cases. Of 233 hospitalized patients (case-fatality rate 14%), >98% had fever, 46% cognitive changes, and 17% abdominal pain or myalgias. Nearly 18% became comatose (*22*). Acute flaccid paralysis was noted, as in the United States in 1999 and later (*23*).

When West Nile virus–infected *Culex* mosquitoes take a blood meal from a susceptible vertebrate, the virus may be incidentally transmitted. Birds serve as amplifying hosts, the degree of amplification depending on avian species, environmental conditions, and other factors. Birds with viremia provide mosquitoes blood meals; these mosquitoes subsequently serve to bridge West Nile virus infection to humans. Responses to recent epizootics and epidemics have improved our understanding of the disease. New competent mosquito vectors are recognized, new human and mammalian symptoms are identified, and new bird species are determined as poor, intermediate, or excellent amplifiers of the virus.

Ludwig et al. examined 437 birds at the Bronx Zoo and Wildlife Conservation Park during the 1999 West Nile virus epizootic and epidemic in New York City (*24*), where virus activity was first recognized in wild and captive birds in the United States. Avian deaths were observed weeks before the first human West Nile virus encephalitis cases. Even though 42% of birds tested were New World birds, 14 (82%) of 17 deaths were in New World birds and 3 (5%) of 57 were in Old World birds, which suggests that birds in the latter group might have had innate immunity by virtue of their ancestral, coevolutionary history with the virus. Diseased birds manifested various symptoms, including abnormal head and neck posture, ataxia, tremors, circling, disorientation, and impaired vision. Most birds with symptoms died.

In Iraq, several mosquito species, including *Culex tritaeniorhynchus*, *Cx. theileri*, and *Aedes caspius* (*25*) have been implicated in West Nile virus transmission. Although mosquitoes in Iraq have not been completely catalogued, it is likely that, as in the United States, other mosquitoes there also serve as vectors of West Nile virus. These mosquitoes are found throughout Iraq, from March to December, and have various larval habitats. Annual spring flooding of the Tigris and Euphrates provides ideal breeding grounds for *Culex* spp. Mosquito species that may have occurred in Babylon are unknown; however, breeding habits must be ancient, and mosquitoes are well known for their proclivity to breed in swamps.

Still, the possibility that West Nile virus killed Alexander is mitigated by the fact that he fell ill in May. Although the virus may have occurred at that time, most recent human cases in Israel occurred in July to September, with only a few cases occurring in June. In temperate areas, West Nile virus infection in humans is seasonal. Amplification occurs in mosquitoes and birds several months before the virus spills over into dead-end hosts. Experimentally infected indigenous mosquitoes showed an intrinsic incubation period of 7 to 14 days at 28°C (*26*). Others have shown that when *Cx. pipiens* mosquitoes were allowed to feed on viremic chicks infected with West Nile virus and incubated at 30ºC virus could be detected 4 days later (*27*). This suggests that maximum virus amplification may not be reached until mid-summer. Iraq’s mean high spring temperature is (29ºC) (*28*), somewhat higher than Tel Aviv’s (24ºC).

Israel has had West Nile virus activity and human cases during the last 3 years, with most human cases not detected until August. Israel is at the same latitude as Iraq and has similar climate. If Iraq also had slightly higher temperatures 2,000 years ago (we will never know this with certainty), onset of disease in humans and birds, including inexplicable avian die-offs, could have occurred earlier in the summer. We reread Plutarch and saw the following passage about Alexander’s entrance into Babylon:  “… when he arrived before the walls of the city he saw a large number of ravens flying about and pecking one another, and some of them fell dead in front of him.” (*29*)

Bird observers (*dāgil işşūri*) were common in Asia Minor at the time. These diviners considered birds as oracles. Greek *Kulturkreis* and Babylonian Alalakh tablets mention auguries based on the behavior of birds, particularly fighting birds, to predict the future (*30*). Plutarch presumably thought it sufficiently noteworthy to record angry or disoriented ravens, although it is impossible to determine whether this event was added later as a necessary metaphoric foreboding of Alexander’s death.

Current geographic distribution of corvids indicates that these likely were ravens (*Corvus corax*) and not crows (*Corvus corone sardonius* or other crow species). No ravens were at the Bronx Zoo in 1999 (T. MacNamara, Wildlife Conservation Society, and pers. comm., 2003). However, in the United States today, New World crows (American crow, *C. brachyrhynchos* and fish crow, *C. ossifragus*) are among the birds most susceptible to fatal West Nile virus infections. One wonders if an influx of migratory birds might have served as reservoirs of West Nile virus and infected ravens in Babylon, causing a massive die-off.

Pathogenicity of West Nile virus for corvids was established 50 years ago. Work et al., assigned by the Rockefeller Foundation to study arboviruses in Egypt, isolated 23 West Nile virus strains from blood samples of febrile children in the Sindbis area and found that the virus caused illness in more children and young adults than in older adults. In addition, and particularly germane to our hypothesis, they isolated West Nile virus from a hooded crow (*Corvus corone sardonius*) for the first time, demonstrating experimental infection of birds with viremia as high as 10^9^, and death rates of 100% (*31*,*32*). During winter, 80% of these crows were seropositive, and the investigators assumed that during transmission season, crow death rates were high. The experimental studies showed that mosquitoes could be infected by feeding on hooded crows with viremia levels as low as 10^3.5^ and could subsequently serve as West Nile virus vectors to humans of any age. This early epidemiologic work provided an early clue in New York City in 1999, when both exotic and domestic birds signaled the introduction of West Nile virus disease to the New World (*33*). Before 1998, the virus was not recognized as an important cause of death in wild birds; therefore, it was surprising to find that the Israeli 1998 strain was the as same that which infected birds at the Bronx Zoo. Ravens dropping dead from the skies likely were also a surprise to Alexander.

## Conclusions

lexander the Great died in late spring in the semi-tropical, urban area of present-day Baghdad. Explanations for his death have included poisoning, enteric and parasitic diseases, influenza, and poliomyelitis. Our diagnosis, as well as previous alternative diagnoses, may be subject to author bias, errors in translation, and a paucity of clinical information. We assumed that he died in late spring in Babylon after a 2-week illness that included fever and signs suggestive of encephalitis. We presumed that diseases now endemic to Iraq were also present in ancient Mesopotamia. Recent scholarly thought has been ingenious and rigorous, given the sparseness of available information. Nonetheless, earlier diagnoses did not include West Nile virus encephalitis. Previous considerations omitted an event that was carefully recorded by Plutarch and which, before 1999, would have been considered irrelevant: the erratic behavior and observable deaths of numerous ravens outside the walls of Babylon. This observation might now be construed as an important clue. If this observation is included as part of Alexander’s illness, West Nile virus encephalitis complicated by flaccid paralysis becomes an alternative diagnosis. It is possible that, in the 3rd century BC, disease caused by West Nile virus arrived in Mesopotamia for the first time in recorded history, killing indigenous birds and an occasional human and causing only incidental febrile illness in many others. Over subsequent centuries the virus may have devolved, becoming less pathogenic for indigenous birds, while retaining its potential as a dangerous human pathogen. This is speculative, but in 1999, a “natural experiment” did occur when this Old World epizootic strain was introduced into the United States. What has been observed in the ongoing North American epizootic and epidemic might be similar to what happened in Babylon many years ago. We now know that unexplained bird die-offs can presage human cases of disease caused by West Nile virus. In 323 BC, a similar event might have been considered an omen of Alexander the Great’s death. In this instance, the oracles would have been correct.
